# Chromosome 1q21 gain is an adverse prognostic factor for newly diagnosed multiple myeloma patients treated with bortezomib-based regimens

**DOI:** 10.3389/fonc.2022.938550

**Published:** 2022-09-14

**Authors:** Xiao Liu, Shuangshuang Jia, Yuping Chu, Biao Tian, Yaya Gao, Chunyan Zhang, Yanhua Zheng, Weijing Jia, Xiangxiang Liu, Ruifeng Yuan, Na Zhang, Juan Feng, Hongjuan Dong, Xiaoli Xin, Ziwei Chang, Zhengcong Cao, Hailong Tang, Guangxun Gao

**Affiliations:** Department of Hematology, Xijing Hospital, The Fourth Military Medical University, Xi’an, China

**Keywords:** multiple myeloma, 1q21, bortezomib, cytogenetics introduction, prognostic factor and survival

## Abstract

Chromosome 1q21 aberration is one of the most common cytogenetic abnormalities in multiple myeloma, and is considered an important prognostic factor. The present study analyzed the clinical relevance and prognostic impact of 1q21 gain in 194 patients with newly diagnosed multiple myeloma treated with bortezomib-based regimens. 1q21 gain was detected in 45.9% (89/194) of patients, and those with 1q21 gain had a worse prognosis. Strikingly, our results showed that excluding the effects of other coinciding genetic anomalies, patients carrying at least four copies of 1q21 had worse survival outcome. Moreover, del(13q) strongly correlates with 1q21 gain, and the coexistence of del(13q) and 1q21 gain plays an important role in reducing PFS and OS times. Therefore, 1q21 gain should be considered a high-risk feature in multiple myeloma patients treated with a bortezomib-based regimen.

## Introduction

Multiple myeloma (MM) is a neoplastic plasma cell disorder characterized by the clonal proliferation of malignant plasma cells in the bone marrow microenvironment. MM accounts for approximately 1% of neoplastic diseases and 13% of hematologic cancers (1). MM is thought to develop through a multistep process, involving genomic instability, epigenetic dysregulation, and interactions within the bone marrow niche during clonal evolution (2). IGH translocations, such as t (4;14), t (6;14), t (11;14), t (14;16), t (14;20), and hyperdiploidy, have been identified as initiation events in this process. Secondary genomic events, such as chromosomal copy number abnormalities, secondary chromosomal translocations, and gene mutations, occur in subclones of MM cells.

Previous studies have confirmed that the clinical heterogeneity of MM depends largely on cytogenetic abnormalities. Based on consensus, trisomies, t (11;14), t (6;14), and a normal karyotype are standard-risk factors associated with relatively good prognosis; t (4;14) is an intermediate-risk factor, while t (14;20), t (14;16), and del(17p) are high-risk factors associated with relatively adverse prognosis (3). 1q21 gain is one of the most common cytogenetic abnormalities in multiple myeloma (4–6). Chromosome 1q21 abnormalities involving the 1q12-23 region are usually complex and tend to become unstable during tumor progression. High-risk copy number gains of 1q21 partly originate from the hypomethylation of 1q12 pericentromeric heterochromatin (7). Nevertheless, the prognostic value of chromosome 1q21 aberrations is still controversial among cytogenetic abnormality studies (8–12).

In this present study, we aimed to explore the prognostic significance of 1q21 gain in patients with newly diagnosed multiple myeloma treated with bortezomib-based regimens to better understand the genetic basis of MM and guide treatment strategies.

## Patients, materials, and methods

### Study design and patients

This was a single-center, retrospective cohort study at the Xijing Hospital of the Air Force Military Medical University. Consecutive patients with *de novo* multiple myeloma receiving bortezomib-based regimens from 2014 to 2021 were included. The median follow-up time was 29 months. The bortezomib-based regimens in our cohort were mainly divided into two categories, one is that the regimen only contains one novel agent bortezomib including VD (bortezomib and dexamethasone) and VCD (bortezomib, cyclophosphamide, and dexamethasone), and the other is that it contains two novel agents bortezomib and immunomodulatory drugs (IMids) including VTD (bortezomib, thalidomide, and dexamethasone) and VRD (bortezomib, lenalidomide, and dexamethasone). All patients were aged at least 18 years and diagnosed according to International Myeloma Working Group (IMWG) criteria.

### FISH analysis

Bone marrow samples were obtained before treatment. All 194 specimens were purified using magnetic-activated cell sorting (MACS) as CD138-positive cells. Post-sorting purity was checked as previously described, and only samples with ≥70% plasma cells after sorting were analyzed. The plasma cell purity was greater than 90% per sample. Then, these specimens were analyzed to detect the following cytogenetic aberrations: 1q21 gain, del(13q), del(17p), t (11;14), t (4;14), t (14;16), and complex karyotype. Complex karyotype was defined as the occurrence of more than two types of chromosome aberration within an abnormal clone by conventional karyotype analysis. The amplification and deletion cutoff values were set at 20%, and samples with translocation present in over 10% of plasma cells were taken into account.

### Outcomes and statistical analyses

The primary objective was to determine the prognostic value of 1q21 gain in MM patients treated with bortezomib-based regimens. Progression-free survival (PFS) and overall survival (OS) times were analyzed as exploratory objectives. The objective response rate (ORR) according to IMWG criteria was assessed as a secondary objective (13). PFS was defined as the time from treatment initiation to the date of documented progression, death, or the last follow-up. OS was defined as the time from the date of treatment initiation to the date of death from any cause or the last follow-up. The Kaplan−Meier method was employed to plot the survival curves, and the log-rank test was used to assess the differences. Logistic regression analysis was used for univariate and multivariate analyses. SPSS version 25.0 (SPSS, Inc) was used for all statistical analyses. Statistical significance was reached if the *p*-value was less than 0.05.

## Results

### Clinical characteristics of 1q21 gain

A total of 194 patients diagnosed with multiple myeloma were enrolled in this study from March 2014 to January 2021, and baseline data are shown in **Table 1**. The median age was 59 years (35–88 years); 45.9% (89) of the patients had advanced ISS III stage, and 17.5% (34) had R-ISS III stage. Three patients were over 80 years of age (1.5%). The median follow-up time was 29 months. The median PFS was 29 months, and the median OS was not reached.

**Table 1:** Patient characteristics.

Characteristics	Overall(*n* = 194)	1q21 gain positive(*n* = 89)	1q21 gain negative(*n* = 105)	*p*
^**a**^ **Median age** **(range) years**	59 (35–88)	59 (35–88)	58 (38–80)	0.429
^**b**^ **Gender (male)**	116 (59.8%)	50 (56.2%)	66 (62.9%)	0.345
^**c**^ **Durie–Salmon stage**				0.218
**I**	21 (10.8%)	6 (6.7%)	15 (14.3%)	
**II**	33 (17.0%)	17 (19.1%)	16 (15.2%)	
**III**	140 (72.2%)	66 (74.2%)	74 (70.5%)	
^**c**^ **ISS stage**				0.003
**I**	35 (18.0%)	7 (7.9%)	28 (26.7%)	
**II**	70 (36.1%)	34 (38.2%)	36 (34.3%)	
**III**	89 (45.9%)	48 (53.9%)	41 (39.0%)	
^**c**^ **R-ISS stage**				<0.001
**I**	23 (11.9%)	5 (5.6%)	18 (17.1%)	
**II**	137 (70.6%)	59 (66.3%)	78 (74.3%)	
**III**	34 (17.5%)	25 (28.1%)	9 (8.6%)	
^**b**^ **M component**				0.175
**IgG**	97 (50.0%)	42 (47.2%)	55 (52.4%)	
**IgA**	47 (24.2%)	27 (30.3%)	20 (19.0%)	
**Others**	50 (25.8%)	20 (22.5%)	30 (28.6%)	
^**d**^ **Light chain**				0.143
**λ**	101 (52.1%)	53 (59.6%)	48 (45.7%)	
**κ**	89 (45.9%)	35 (39.3%)	54 (51.4%)	
**Others**	4 (2.1%)	1 (1.1%)	3 (2.9%)	
^**a**^ **Marrow plasma cell (%) (range)**	36.8 (2.0–94.8)	40.00 (10.4–94.8)	31.60 (2.0–89.6)	0.062
^**b**^ **Peripheral plasma cells**	35 (18.0%)	20 (22.5%)	15 (14.3%)	0.140
^**a**^ **Hemoglobin (g/L) (range)**	93.0 (42.0–165.0)	93.0 (42.0–143.0)	94.0 (46.0–165.0)	0.091
^**a**^ **Albumin (g/L) (range)**	34.75 (13.40–51.40)	33.60 (13.40–45.00)	36.30 (18.60–51.40)	0.004
^**a**^ **Calcium (mmol/L) (range)**	2.24 (1.74–3.47)	2.27 (1.74–3.47)	2.21 (1.77–3.37)	0.707
^**a**^ **β_2_-MG (mg/L) (range)**	5.01 (1.37–87.4)	6.09 (2.04–44.3)	3.90 (1.37–87.4)	0.266
^**b**^ **LDH high level**	31 (16.0%)	20 (22.5%)	11 (10.5%)	0.023
^**b**^ **Extramedullary lesions**	93 (47.9%)	46 (51.7%)	47 (44.8%)	0.336
^**b**^ **Novel therapy**				0.413
**BTZ + IMids-based**	89 (45.9%)	38 (42.7%)	51 (48.6%)	
**BTZ-based**	105 (54.1%)	51 (57.3%)	54 (51.4%)	
^**b**^ **ASCT accepted**	21 (10.8%)	11(12.4%)	10 (9.5%)	0.526

Ig, immunoglobulin; ISS, International Staging System; R-ISS: Revised International Staging System; β_2_-MG, β_2_-microglobulin; LDH, lactate dehydrogenase; BTZ: Bortezomib.

All 180 patients. (B) Patients with/without the gain of 1q21.

Statistical tests used:

a t-test.

b M-L χ^2^ test.

c Kruskal–Wallis test.

d Fisher test.

### Association between 1q21 gain and clinical and biologic parameters

In the present study, 1q21 gain was detected in 45.9% (89/194) of MM patients. Compared to patients without 1q21 gain, 1q21 gain patients demonstrated the following clinical characteristics (**Table 1**): (1) More advanced ISS (*p* = 0.003) and R-ISS (*p*<0.001) stages, (2) lower serum albumin levels (ALB: *p* = 0.004), and (3) higher lactic dehydrogenase levels (*p* = 0.023). There were no significant differences between the 1q21 gain and non-1q21 gain groups in other clinical baselines, including sex, DS stage, heavy and light chain, myeloid and serum plasma cell, calcium, β2-microglobulin, extramedullary disease, and receipt of ASCT (*p* > 0.05), and there were also no significant differences in treatment regimens (*p* = 0.413) (**Table 1**).

### 1q21 gain is an independent risk factor for newly diagnosed myeloma patients

In our cohort, we used univariable logistic regression to evaluate the risk factors of survival outcome in newly diagnosed multiple myeloma patients. The results showed that among all variables, only 1q21 copy number, β2-microglobulin, t (4;14), and presence of peripheral plasma cells were the risk factors (*p*<0.05) affecting the survival outcome. Finally, we picked up these four variables to perform multivariable logistic regression analysis. The results showed that 1q21 copy number was an independent risk factor for newly diagnosed multiple myeloma (**Table 2**).

**Table 2:** Univariable and multivariable logistic regression of survival outcome.

	Univariable analysis			Multivariable analysis		
	OR	95% CI	*p*	OR	95% CI	*p*
**1q21 copy number**						
**Normal**	–	–	–	–	–	–
**Gain1q (3 copies of 1q21)**	3.598	1.277–10.135	0.015	3.482	1.148–10.560	0.028
**Amp1q (≥4 copies of 1q21)**	5.009	2.109–11.899	<0.001	3.876	1.549–9.699	0.004
**β_2_-MG**	1.045	1.010–1.081	0.012			
**t (4;14)**	6.259	2.539–15.428	<0.001	5.314	1.858–15.198	0.002
**Peripheral plasma cell**	2.889	1.297–6.435	0.009	3.177	1.210–8.345	0.019

### Association between 1q21 gain and other cytogenetic abnormalities

In our assessment of concurrent chromosome aberrations, del(13q) and del(17p) were detected in 59.6% (*p* = 0.014) and 10.1% (*p* = 0.891) of patients with 1q21 gain, respectively; in addition, t (4;14), t (11;14), and t (14;16) were detected in 18.0% (*p* = 0.029), 15.7% (*p* = 0.381), and 3.4% (*p* = 0.662) of these patients, respectively. The incidence of other cytogenetic abnormalities, including del(17p), del(13q), and all types of IgH translocation, was 73.0% in 1q21 gain cases and 54.3% in non-1q21 gain cases (*p* = 0.007). Importantly, a complex karyotype was more common in patients with 1q21 gain than in those without (*p*<0.001). In conclusion, a significant correlation between 1q21 gain and del(13q), t (4;14), and other cytogenetic aberrations or complex karyotypes was observed (**Table 3**).

**Table 3:** Associations between 1q21 gain subgroup and cytogenetic abnormalities.

	Overall(*n* = 194)	1q21 gain positive(*n* = 89)	1q21 gain negative (*n* = 105)	*p*
^**a**^ Del(13q)	97 (50.0%)	53 (59.6%)	43 (41.9%)	0.014
^**a**^ Del(17p)	19 (9.8%)	9 (10.1%)	10 (9.5%)	0.891
^**a**^ t (11;14)	26 (13.4%)	14 (15.7%)	12 (11.4%)	0.381
^**a**^ t (4;14)	24 (12.4%)	16 (18.0%)	8 (7.6%)	0.029
^**b**^ t (14;16)	5 (2.6%)	3 (3.4%)	2 (1.9%)	0.662
^**a**^ Other cytogenetic aberration	122 (62.9%)	65 (73.0%)	57 (54.3%)	0.007
^**a**^ Complex karyotype	84 (43.3%)	65 (73.0%)	19 (18.1%)	<0.001

Other cytogenetic aberration was defined as del(17p), del(13q), and all type of IgH translocation by FISH.

Complex karyotype was defined as the occurrence of more than two types of chromosome aberrations within an abnormal clone by conventional karyotype analysis.

Statistical tests used:

a M-L χ^2^ test.

b Fisher test.

### Survival analysis of patients with 1q21 gain

**Figure 1:**
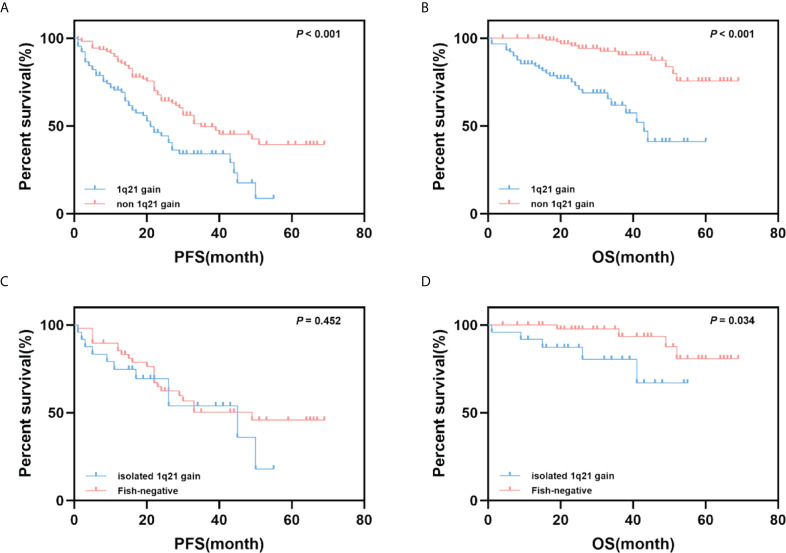
Impact of 1q21 gain on PFS and OS. **(A, B)** Kaplan–Meier curves are shown for PFS and OS for patients with 1q21 gain and without 1q21 gain. **(C, D)** Kaplan–Meier curves are shown for PFS and OS for patients with 1q21 gain only and with no cytogenetic abnormalities.

A survival analysis was performed to assess the impact of 1q21 gain on PFS and OS in MM patients. Patients with 1q21 gain had significantly shorter PFS and OS times than those without 1q21 gain (median PFS: 21 months *vs*. 35 months, *p*<0.001; median OS: 43 months *vs*. NR, *p*<0.001). Given that 1q21 gain is more likely to coexist with other cytogenetic abnormalities, especially high-risk cytogenetics, we further compared patients with isolated 1q21 gain and those who were FISH-negative. The analysis showed that isolated 1q21 gain was an adverse prognostic factor of OS (*p* = 0.034), and there were no significant differences in PFS between patients with isolated 1q21 gain and those who were FISH-negative (**Figure 1**).

### Prognostic value of 1q21 gain at different copy numbers

**Figure 2:**
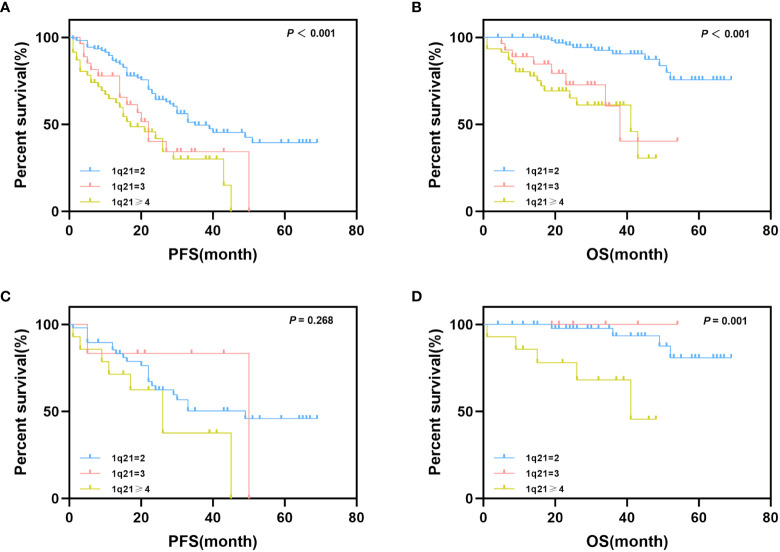
Impact of 1q21 gain copy numbers on PFS and OS. **(A, B)** Kaplan–Meier curves are shown for PFS and OS for patients with different copy numbers of 1q21 gain. **(C, D)** Kaplan–Meier curves are shown for PFS and OS for isolated 1q21 gain patients and FISH-negative patients with different copy numbers of 1q21 gain.

To further analyze the effect of 1q21 gain on the survival and prognosis of patients with MM, we grouped patients according to different 1q21 copy numbers. Survival analysis results showed that patients with a normal copy number of 1q, Gain1q, and Amp1q had a median PFS of 35, 22, and 17 months, respectively (*p*<0.001), and the median OS of the three groups was NR, 38 months, and 41 months, respectively (*p*<0.001) (**Figures 2A, B**). Obviously, over three copies of 1q led to poor prognosis; however, there was no significant difference between Gain1q and Amp1q patients.

As mentioned above, we also considered the possible effects of coexisting cytogenetic abnormalities; thus, we further explored the impact of 1q21 copy number on prognostic data in isolated 1q21 gain and FISH-negative patients. Patients were grouped according to the protocol described above. The median PFS times of normal copy number of 1q, Gain1q, and Amp1q were 49, 50, and 26 months, respectively (*p* = 0.268), and the median OS times were NR, NR, and 41 months, respectively (*p* = 0.001) (**Figures 2C, D**).

### Gain of 1q21 and response rate

Only 186 enrolled patients had evaluable results for the best treatment response after bortezomib-based chemotherapy. Among patients with 1q21 gain (*n* = 89), the ORR was 74.2%, with the following distribution: CR 30.3%, VGPR 29.3%, and PR 14.6%. Among patients without 1q21 gain (*n* = 105), 87.6% achieved ORR, including 29.5% CR, 36.2% VGPR, and 21.9% PR. Patients without 1q21 gain had a higher ORR rate than patients with 1q21 gain (*p* = 0.027). Similarly, the patients with isolated 1q21 gain had a lower ORR rate than FISH-negative patients (63.6% *vs*. 89.1%, *p* = 0.020) (**Table 4**).

**Table 4:** Treatment response rate.

	1q21 gain Negative(*n* = 105)	1q21 gain Positive(*n* = 89)	*P*	FISH-negative(*n* = 46)	1q21 gain only (*n* = 22)	*P*
**ORR**	92 (87.6%)	66 (74.2%)	0.027	41 (89.1%)	14 (63.6%)	0.020
**CR**	31 (29.5%)	27 (30.3%)	0.798	15 (32.6%)	10 (45.5%)	0.304
**≥VGPR**	69 (65.7%)	53 (59.6%)	0.516	30 (65.2%)	11 (50.0%)	0.230

ORR, overall response rate; CR, complete response; VGPR, very good partial response; PR, partial response.

### Prognostic value of 1q21 gain combined with other cytogenetic abnormalities

As mentioned above, 1q21 gain and other cytogenetic abnormalities significantly correlated; thus, we further explored the combined effects of 1q21 gain and other cytogenetic abnormalities on patient outcomes. Del(17p), t (4;14), and t (14;16) were considered high-risk cytogenetic abnormalities (HRCAs) according to the IMWG standard (14). Patients were divided into four groups according to HRCAs and 1q21 gain in the subsequent analysis. No significant differences in PFS were observed in patients with HRCAs versus FISH-negative patients; however, patients with 1q21 gain showed shorter PFS times than HRCA patients (median PFS: 20 *vs*. 45 months, *p* = 0.044). Regarding OS, the adverse impact of 1q21 gain was enhanced when it coexisted with HRCAs (median OS: NR *vs*. 38 months, *p* = 0.045).

**Figure 3:**
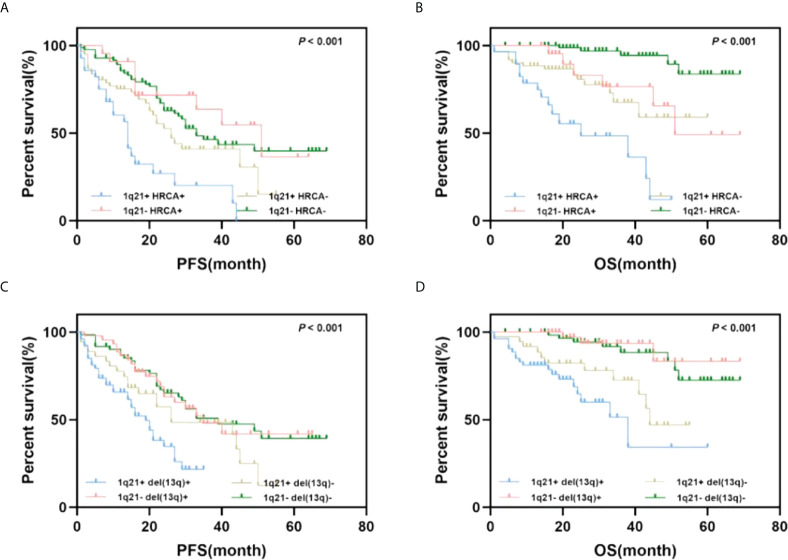
Impact of 1q21 gain coexisting with other cytogenetics on PFS and OS. **(A, B)** Kaplan–Meier curves are shown for PFS and OS for patients with or without 1q21 gain and high-risk cytogenetics abnormalities. **(C, D)** Kaplan–Meier curves are shown for PFS and OS for patients with or without 1q21 gain and del(13q).

In addition, we analyzed the synergistic effect of 1q21 gain and del(13q), which was not an independent predictor of poor prognosis mentioned above (15). The results showed that PFS and OS among the four groups were significantly different (*p* < 0.001). Importantly, patients with del(13q) only [1q21- del(13q)+] had better OS times than those in the other three groups (NR *vs*. 44 months *vs*. NR *vs*. 38 months). The median OS of patients with 1q21 gain and del(13q) [1q21+ del(13q)+] was shorter than that of the OS of those with 1q21 gain only [1q21+ del(13q)-](38 *vs*. 44 months, *p* = 0.138); however, the statistical analysis revealed that the difference was not significant (**Figure 3**).

## Discussion

We report a real-world retrospective study of the prognosis and efficacy of 1q21 gain in patients with newly diagnosed multiple myeloma who received bortezomib-based chemotherapy. We found that 1q21 gain is common (45.9%) at diagnosis in multiple myeloma, and this aberration is associated with later ISS stage levels, lower serum albumin levels, and elevated LDH concentrations, consistent with previous research results (3, 8, 16). Moreover, we further demonstrated the prognostic risk value of 1q21 gain in PFS and OS in MM patients. 1q21 gain could significantly result in an adverse outcome when the effects of other cytogenetic abnormalities were excluded.

In recent years, 1q21 gain has been identified as a potential poor prognostic factor. Nahi et al. (17) and Saxe et al. (4) found that even in the era of new drugs, 1q21 gain still led to poor PFS and OS. Shah et al. (18) and Mohan et al. revealed that CD38 monoclonal antibody and ASCT could not reverse the poor prognosis associated with 1q21 gain. According to the latest Mayo guide, MM patients with gain (1q21) are at a higher risk for progression, including those with MGUS, SMM, and multiple myeloma (3). A multivariate analysis conducted by Abdallah et al. revealed that 1q21 gain was an independent risk factor for OS in MM patients (16). However, 1q21 gain is not included in the stratification of the European myeloma consensus (19), and some studies failed to demonstrate the relationship between 1q21 gain and adverse prognosis, although some of the patients received conventional chemotherapies (12, 20, 21). Our results demonstrated that 1q21 gain is a poor prognostic factor in MM patients and is associated with poor clinical features, such as a high concentration of LDH.

In addition, the importance of 1q21 copy number in MM patient prognosis is contradictory. Neben et al. showed that compared with a normal copy number of 1q21, a copy number of three has a marginal negative effect, and having more than three copies significantly reduces PFS and OS times (22). Schmidt et al. also demonstrated that only copy numbers greater than or equal to 4 led to a poor prognosis (10). In contrast, Abdallah et al. observed similar prognostic effects between three and more than four 1q21 copies (16). Consistently, a Chinese study also failed to prove the relationship between different 1q21 copy numbers and prognosis (5). Moreover, Locher demonstrated that three or more than three copies of 1q21 are both adverse prognostic factors, and the effect of more than three copies is more obvious (23). We set the cutoff value of 1q21 gain to 20% and found that the PFS and OS of Gain1q and Amp1q were significantly shorter than those with normal copies of 1q21, but the effect of Gain1q and Amp1q on prognosis was similar. However, excluding the coexistence of other genetic abnormalities, we found that the prognosis of Amp1q patients was significantly worse than that of patients with a normal copy number or Gain1q.

Multiple myeloma patients often carry more than one cytogenetic abnormality, and recent studies have indicated that the coexistence of many abnormalities is a crucial prognostic indicator. For instance, Boyd et al. demonstrated that 1q21 gain, del(17p), and IgH translocation often coexist, and the accumulation of these adverse abnormalities is associated with gradually worsening survival outcomes (24). Pawlyn also found similar results (25). In contrast, Kumar et al. found that 1q21 chromosomal trisomy ameliorated the adverse effects of t (4;14), t (14;16), and t (14;20) (11). We found that 1q21 gain was associated with del(13q) and t (4;14), and patients with 1q21 gain often had complex karyotypes. Further analysis showed that the coexistence of 1q21 with other genetic abnormalities leads to a worse prognosis. Moreover, we found that del(13q) strongly correlates with 1q21 gain and that the coexistence of del(13q) and 1q21 gain plays an important role in reducing PFS and OS times.

Previous studies have shown that bortezomib-based regimens cannot overcome the adverse effects of 1q21 (10, 17). We further analyzed the response of enrolled patients to this treatment protocol. Patients with only 1q21 gain had a lower ORR. However, if the effects of coexisting genetic abnormalities are not excluded, it cannot be confirmed that patients with 1q21 gain have worse treatment responses. Coexisting genetic abnormalities may reverse the role of 1q21 in bortezomib resistance.

Our study has the standard limitations of retrospective studies, including inadequate number of cases and selection bias. Moreover, only a portion of the enrolled patients had 1q21 copy number aberrations, and the sample size used for the statistical analysis after excluding those with coexisting genetic abnormalities was small. In addition, the heterogeneity of the treatment regimens also needs to be considered; thus, our results need to be verified with further follow-up and prospective studies.

In conclusion, our study demonstrated the importance of 1q21 gain in myeloma patients treated with bortezomib-based regimens. 1q21 gain was an independent prognostic risk factor for PFS and OS and led to worse treatment responses. Routine testing should include FISH for 1q21 gain, and patients with this abnormality should be considered for alternative treatments and new drugs might improve their prognosis.

## Data availability statement

The original contributions presented in the study are included in the article/supplementary material. Further inquiries can be directed to the corresponding authors.

## Ethics statement

This study was reviewed and approved by Ethics Committee of Xijing Hospital of Air Force Medical University. The patients/participants provided their written informed consent to participate in this study.

## Author contributions

XL, SJ and YC analyzed the majority of data and prepared figures. XL wrote the manuscript. BT, YG, CZ, YZ, WJ and XXL collected the data. RY, NZ, JF and HD collated the data. XX, ZWC and ZCC assisted in data sorting and analysis. GG and HT conceived the study, designed the study, evaluated data, and revised the manuscript. All authors contributed to the article and approved the submitted version.

## Funding

This work was supported by the National Natural Science Foundation of China (82100218, 81970190, and 81900207).

## Acknowledgments

The authors thank the support from all the medical and nursing staff of the Department of Hematology, Xijing Hospital, Air Force Medical University, Xi’an, Shaanxi, China.

## Conflict of interest

The authors declare that the research was conducted in the absence of any commercial or financial relationships that could be construed as a potential conflict of interest.

## Publisher’s note

All claims expressed in this article are solely those of the authors and do not necessarily represent those of their affiliated organizations, or those of the publisher, the editors and the reviewers. Any product that may be evaluated in this article, or claim that may be made by its manufacturer, is not guaranteed or endorsed by the publisher.
